# The molecular portrait of *in vitro *growth by meta-analysis of gene-expression profiles

**DOI:** 10.1186/gb-2005-6-8-r65

**Published:** 2005-07-27

**Authors:** Rickard Sandberg, Ingemar Ernberg

**Affiliations:** 1 Microbiology and Tumor Biology Center (MTC), Karolinska Institutet, S-171 77 Stockholm, Sweden; 2 Department of Biology, Massachusetts Institute of Technology, Cambridge, MA 02139, USA

## Abstract

**Background:**

Cell lines as model systems of tumors and tissues are essential in molecular biology, although they only approximate the properties of *in vivo *cells in tissues. Cell lines have been selected under *in vitro *conditions for a long period of time, affecting many specific cellular pathways and processes.

**Results:**

To identify the transcriptional changes caused by long term *in vitro *selection, we performed a gene-expression meta-analysis and compared 60 tumor cell lines (of nine tissue origins) to 135 human tissue and 176 tumor tissue samples. Using significance analysis of microarrays we demonstrated that cell lines showed statistically significant differential expression of approximately 30% of the approximately 7,000 genes investigated compared to the tissues. Most of the differences were associated with the higher proliferation rate and the disrupted tissue organization *in vitro*. Thus, genes involved in cell-cycle progression, macromolecule processing and turnover, and energy metabolism were upregulated in cell lines, whereas cell adhesion molecules and membrane signaling proteins were downregulated.

**Conclusion:**

Detailed molecular understanding of how cells adapt to the *in vitro *environment is important, as it will both increase our understanding of tissue organization and result in a refined molecular portrait of proliferation. It will further indicate when to use immortalized cell lines, or when it is necessary to instead use three-dimensional cultures, primary cell cultures or tissue biopsies.

## Abstract 2

A meta analysis comparing 60 tumor cell lines, 135 normal tissue samples and 176 tumor tissue samples in humans shows significant differential expression between cell lines and tissues of around 30% of the 7,000 genes analyzed.

## Background

How different are cells grown *in vitro *from cells that are part of a tissue? Human tissues and tumors are complex and heterogeneous as they are composed of different cell types that influence each other through paracrine signaling pathways and interactions with extracellular matrix (ECM). Cell lines on the other hand consist of a more or less clonal cell populations that lack interactions with other cell types and interact with an artificial support such as plastic. Cell adaptation to *in vitro *microenvironments have probably involved recalibrations of many cellular pathways through genetic alterations [1], transcriptional alterations [2], different post-transcriptional regulation [3] and changed signaling networks [4]. Thus, the degree to which cell lines are representative of the specific cell types they were derived from varies [5,6]. Furthermore, among cell lines established for *in vitro *growth there is an overwhelming bias for tumor-derived cells. It has been very hard to establish non-transformed cells for long-term *in vitro *growth. Detailed comparisons of the genotypic and phenotypic characteristics of *in vitro *grown cells with a panel of normal and tumor tissues may reveal how cell lines have adapted to *in vitro *environments. Moreover, comparisons of cell lines with both tumors and the normal tissues they were derived from are needed to assess how well they represent their tissue of origin and which of their features may have been acquired in *vitro*.

Analyses of mRNA expression levels using DNA microarrays have contributed to an increasingly detailed understanding of patterns of gene expression in different tissues [7,8] and also how *in vitro *selection and adaptation affect basic cellular processes. So far, these studies have been focused on single cell types. Cell lines from colon [9], breast [10], lymphoma [11], leukemia [2], and lung origin [12] have been compared to their corresponding *in vivo *malignancies. These studies have consistently demonstrated that different cell lines of the same tissue origin are more similar to each other than to the tumors they derived from. From these gene-expression studies, it has also been repeatedly shown that genes associated with proliferation [2,10,11] and ribosomal activity [9] are upregulated in cell lines. However, no study so far has addressed the issue of whether the same genes are perturbed by the *in vitro *environment in cell lines derived from tumors of different tissue origins, that is, if there may be an '*in vitro *expression profile'.

Developing meta-analytical tools for comparing gene-expression data generated in different studies and laboratories is important. Some meta-analysis of gene-expression profiles of multiple tumors and normal tissues have been pursued, identifying common upregulated genes in neoplastic transformation and in relation to tumor differentiation status [13]. Moreover, a collection of gene-expression data from different tumor types has been used to identify upregulated or repressed modules of genes with coherent expression profiles in specific tumors [14]. In both these studies, gene-expression data was gathered from multiple platforms and laboratories, although the data were analyzed independently (that is, for each dataset separately). In the first study, the expression levels in each array were normalized independently to unit length (a median expression of zero and a standard deviation of one) [13]. In the second study, each gene was subtracted by the mean expression level across the samples in each dataset, respectively [14]. Subsequently, genes which were consistently up- or downregulated could be identified in comparisons within multiple datasets [13].

In this study, we describe a cross-site approach to quantitatively integrate gene-expression profiles from three laboratories [15-17] comprising 60 cell lines and 311 tissue samples. We integrated gene-expression data from cell lines derived from tumors of nine different tissue-origins (NCI60 cell lines) with two large gene-expression datasets of human tissues and human tumors. All these studies used the same platform and array-type (Affymetrix Hu6800). Using a meta-analysis we defined the transcriptional changes observed in all cell lines compared to both normal and tumor tissues independent of tissue origin. The cell lines showed statistically significant differential expression of approximately 30% of the approximately 7,000 genes investigated. Among the upregulated genes we consistently found - not surprisingly - many genes involved in macromolecular turnover, cell-cycle progression, energy metabolism, and histone modifications. Adhesion molecules and membrane signaling proteins were enriched among the downregulated genes, a possible consequence of the disrupted tissue organization *in vitro*. The origin-independent transcriptional alterations defined in this study are probably the consequence of the *in vitro *adaptation and selection. As such, our data will be important to improve our understanding of the biological consequences of *in vitro *growth and thus how well cell lines correspond to the *in vivo *tissues and tumors.

## Results

### Normalization of gene-expression profiles from multiple sources

To study the expression signature of *in vitro *growth, we collected gene-expression profiles from 60 cancer cell lines [15], 135 normal tissue samples [16,17] and 176 tumor tissue samples [16] generated using the same Affymetrix Hu6800 array platform (dataset I). The cell lines were derived from nine different tumor types, the normal tissues samples 19 tissues and the tumor samples from 13 different tissues (see Materials and methods). As a control, we also used gene-expression data from an independent study, in which both cell lines and tissues were profiled within the same study [18] using Affymetrix HGU95A arrays (dataset II). Dataset II was more limited, however, as 21 of the 25 cell-line samples were of lymphoid origin. Together, these two datasets (Table 1) were considered as well suited to systematically evaluate how cell lines in general approximate their tissues of origin and thus their resulting validity as biological model systems.

**Figure 1:**
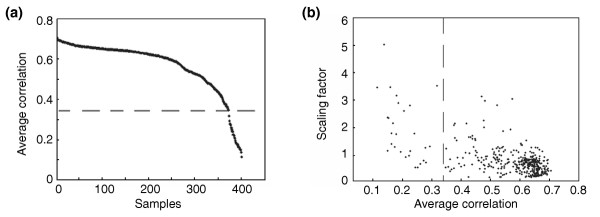
Identification of outlier samples by correlation analysis and scalar factors. **(a) **Plotting the average correlation for each sample from pairwise comparisons to all other samples (*y*-axis). The samples were sorted according to their average correlation (*x*-axis). We used an average correlation of 0.34 as a cutoff (marked with a dashed line). **(b) **Comparison of the average correlation (*x*-axis) with the scalar factor used in the global scaling procedure (*y*-axis). Many of the samples with low average correlations had been rescaled using high scaling factors, indicating that they might have had poor hybridizations. Again, the dashed line displays the average correlation cutoff.

**Figure 2:**
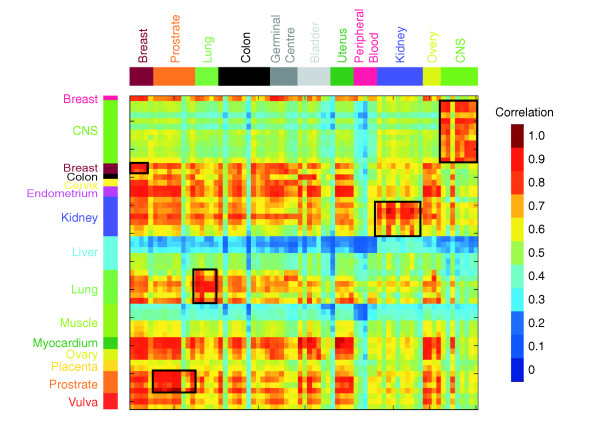
Correlation matrix between all normal samples from two studies. The gene-expression profiles of each normal tissue sample were compared to all other normal tissue samples from the other dataset by measuring the correlation across all genes. The normal samples from Hsiao *et al*. [17] are presented along the *y*-axis and samples from Ramaswamy *et al*. [16] along the *x*-axis. The correlation matrix displays each pairwise comparison and each entry is color-coded according to the scale bar to the right of matrix. Black rectangles highlight correlation values between the samples from the same tissues in the two different datasets.

It must be emphasized that comparing gene-expression data from different laboratories may introduce different biases resulting from different experimental conditions and protocols. To quantitatively compare gene-expression profiles from different studies, we rescaled all samples using the global scaling algorithm (see Materials and methods). We investigated each sample after the rescaling procedure to check whether any samples were of questionable quality by computing its average correlation to all other samples. This analysis step served two purposes: first, to investigate how similar were the gene-expression patterns of the biological replicates; second, to verify that samples of the same tissues in the different datasets were more similar to each other than to other tissues. Overall, the average correlations between samples of different tissue origins were between 0.5 and 0.6. Certain samples, however, were found to have an average correlation to other samples as low as 0.15 (Figure 1a). These samples with low average correlation also had higher scaling factors (Figure 1b), indicating that they had lower signals on the chip. This could be a result of a less successful hybridization, and it is likely that our rescaling procedure worked less efficiently for these samples. Therefore, we removed the 28 samples with an average correlation of less than 0.34 (Figure 1). The removed samples were of diverse tissue origins and the low average correlation observed for these samples was not an effect of being a single sample from a specific tissue. We used the gene-expression profiles of the same normal tissues that were present in two of the datasets [15,16] as an initial evaluation of the rescaling procedure. The expression profiles from the same tissues should be more similar to each other than to samples from other tissues, independently of the laboratory in which the data were generated. We compared the correlation between the 59 normal samples from Hsiao *et al*. [17] to the 91 normal samples from Ramaswamy *et al. *[16]. The matrix of correlations is presented in Figure 2. Gene-expression profiles of the same tissues gathered in the two laboratories showed in general higher correlations, indicating that tissue-specific differences within each dataset were larger than a possible systematic difference between the two datasets. There were, however, high correlations between gene-expression profiles of hormone-related tissues (for example, breast, ovary and uterus) both within and between datasets.

### Validation of the quantitative comparison across datasets

**Figure 3:**
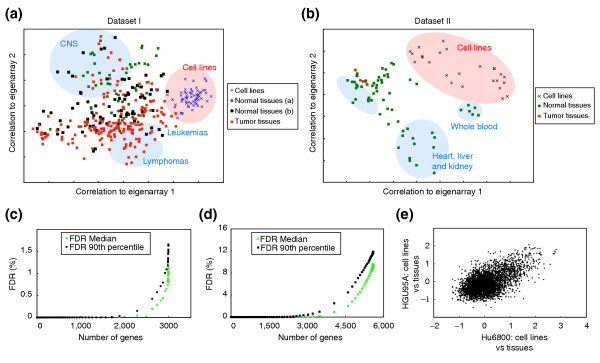
The gene-expression profiles of cell lines compared to normal and tumor tissues. **(a) **Projection of each sample in dataset I into SVD space drawn by the correlation of each sample to SVD eigenarray 1 (*x*-axis) and 2 (*y*-axis). The normal tissue samples of CNS origin from two laboratories (green squares, Hsiao *et al *[17]; black squares, Ramaswamy *et al*. [16]) were overlapping, as well as the tumor tissue samples (red squares, Ramaswamy *et al*. [16]). The cell lines were separated from tissue samples by the first SVD eigenarray. Samples of lymphoma and leukemia origin were also separated in the SVD analysis. **(b) **Projection of each sample in dataset II into the SVD space drawn by the correlation of each sample to SVD eigenarray 1 (*x*-axis) and 2 (*y*-axis). The cell lines (crosses) were separated from tissue samples. Whole blood samples were distinctly clustered close to the cell lines. **(c) **Other separation of normal samples. Significance analysis of microarrays (SAM) was used to identify differentially expressed genes between cell line and tissue samples in dataset I. The number of statistically significant genes (*x*-axis) as a function of the median and 90th percentile of the FDR (*y*-axis) estimated based on 1,000 permutations. **(d) **SAM analysis of cell line versus tissue samples in dataset II. Identical parameters as in (c). **(e) **Plot of the degree of differential expression between cell lines and tissues for each gene in dataset I (*x*-axis) versus dataset II (*y*-axis) respectively. The degree of differential expression was measured using the signal-to-noise metric [23].

Singular value decomposition (SVD) has been successfully used to investigate the fundamental patterns in gene-expression data [19,20]. We analyzed our merged gene-expression data (dataset I) using SVD to asses the fundamental patterns within the data, and in particular the similarities between the expression data from the different laboratories. We projected each sample into a SVD subspace by calculating the correlation between the expression profiles of each array and the two eigenarrays (derived from the SVD), respectively (Figure 3a). Because the first two eigenarrays are associated with the two largest singular values [19,21], this procedure captures the largest variability inside the gene-expression data into a two-dimensional plot. Importantly, the gene-expression profiles of normal tissue samples from the two different studies were overlapping after the SVD projection. Moreover, normal tissue and tumor tissue samples of CNS origin, from the two different laboratories, were in proximity to each other in SVD subspace (Figure 3a). Therefore, laboratory-dependent separation of the tissue samples was not observed. However, the cell lines were distinctly separated (Figure 3a). This could reflect either a technical artifact in the merging of only the gene-expression data of the cell lines, or that the cell lines have very different gene-expression profiles compared to tissues.

Therefore, we performed the identical analysis of dataset II (the validation dataset) comprising both cell lines and tissue samples within the same study. Using the identical SVD procedure, cell lines were again separated from tissues in their correlation with the two first eigenarrays (Figure 3b). This excluded the possibility that the cell line versus tissues distinction in dataset I was a technical artifact. Moreover, the separation of cell lines from tissue samples was captured by the first eigenarray in both datasets demonstrating that this difference was the largest in the gene-expression data. Hierarchical clustering of the gene expression in datasets I and II, were also found to repeatedly separate all cell lines from normal and tumor tissues (data not shown).

### Identification of origin-independent transcriptional alterations *in vitro*

We next sought to estimate the number of genes that were specifically up- or downregulated in cell lines and responsible for the distinct separation of cell lines from tissue samples. We used significance analysis of microarrays (SAM) [22] to identify the number of genes with statistically significant differential expression as a function of the false discovery rate (FDR). In dataset I, using conservative criteria, we identified 1,500 genes with an estimated FDR of zero, and 2,900 genes at a FDR of 1% (Figure 3c). For example, at a FDR of 1% only 29 false positives are estimated out of the 2,900 genes identified. In dataset II we identified 1,800 genes at a FDR of zero and 3,400 genes at a FDR of 1% (Figure 3d). In total, using a FDR of 1%, we identified 41% of the genes as differentially expressed between cell lines and tissues in dataset I and 29% in dataset II respectively. To investigate the generality of our results, we investigated whether the identical genes were identified as up- or downregulated in cell lines in dataset I and II despite the sample and platform differences. Of the 2,000 most differentially expressed genes in dataset I, we found corresponding probe sets for 1,476 of the genes on the HGU95A arrays (635 upregulated and 841 downregulated genes) using a recently published map [23]. We confirmed the upregulation of 399 genes (63% of the genes; *p *< 4e-70, Fisher's exact test) and 176 (21% of the genes; *p *< 1e-7, Fisher's exact test) of the downregulated genes in cell lines by identifying the intersection with the genes with statistically significant differential expression in dataset II (FDR of 1%). The list of genes found to be differentially expressed in both datasets is found in Additional data file 1. Second, we also compared the score of differential expression for all genes in both datasets (Figure 3e). A correlation coefficient of 0.33 between the degree of differential expression in dataset I and II was observed, even though they are generated using two different Affymetrix arrays and the sample origins were diverse. Again, this demonstrated that the results obtained by comparing the cell lines to normal and tumor tissues in dataset I were not due to technical artifacts.

### Classification of samples based upon the *in vitro *signature

To further validate that the gene-expression differences between cell lines and tissues identified in both dataset I and II (399 upregulated and 176 downregulated genes) represent true transcriptional alterations associated with long-term cultured cell lines, we evaluated the ability to classify samples on the basis of these genes (Materials and methods). First, as a control, we classified each sample in dataset I and II into either 'cell line' or 'tissue'. The accuracy of the classification was 99% and 100% respectively (Table 2). Second, we classified each sample in three additional datasets [8,12,24], again with high accuracy (Table 2). Plots of the distributions of scores for each dataset can be found in Additional data file 2.

### Features of the *in vitro *gene-expression signature

**Figure 4:**
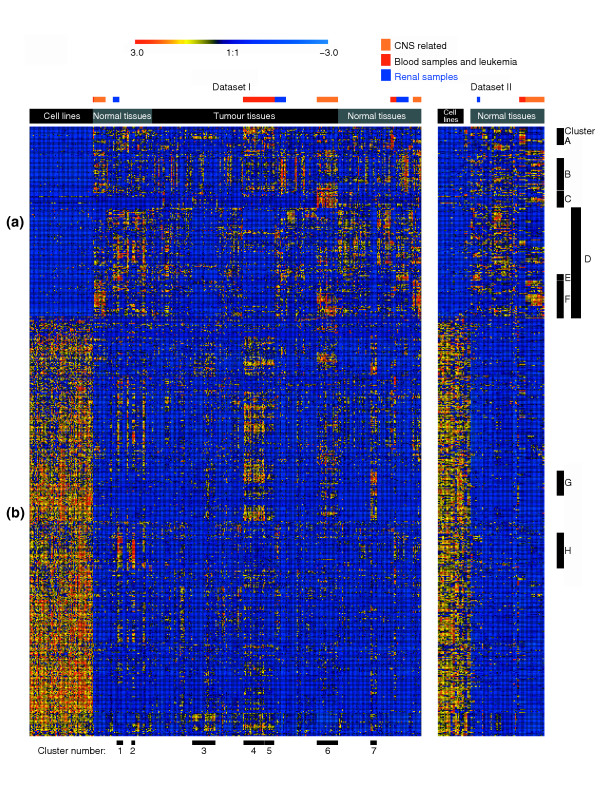
The gene-expression signature of *in vitro *growth. All genes found to be differentially expressed between cell lines and tissues across two dataset I and II (576 genes) were subject to hierarchical clustering (average linkage and Euclidean distance metric) using the Genesis software [43]. Before clustering, all genes were normalized to an average expression level of zero and a standard deviation of one (that is unit length). Above the cluster image, samples are labeled as cell lines, normal tissues and tumor tissues (except for the primary cultures and FACS-sorted cells in datasets II that were not annotated). **(a) **Top part of the cluster presents the genes found to be downregulated *in vitro*. These genes were not detected *in vitro *and were often only expressed in a subset of tissue samples. It is likely that these genes represent downregulated tissue markers from the respective tissues. **(b) **In contrast, genes found to be upregulated *in vitro *were highly expressed in all cell lines, while occasionally expressed in a few tissue samples. Specific clusters of genes in (a) and (b) are annotated on the right of the cluster image (clusters A to H). Specific groups of samples are annotated in color above the cluster image and by number below the cluster image (cluster numbers 1 to 7). Cluster number 1, kidney and liver samples; cluster number 2, lung and muscle; cluster number 3, lymphomas; cluster number 4, leukemias (ALL); cluster number 5, leukemias (AML); cluster number 6, CNS tumors (medullablastoma and glioblastoma); cluster number 7, germinal center cells.

We observed a qualitative difference in the expression patterns of the up- and downregulated genes in cell lines that might explain the higher degree of confirmation of upregulated genes in dataset II. Figure 4 shows the general trends in the expression of differentially expressed genes in both dataset I and II across cell lines and tissues. The upregulated genes were highly expressed all cell lines and in general expressed in lower amounts in tissue samples (Figure 4b; some exceptions are discussed below). Genes found to be downregulated in cell lines were low in all cell lines, but highly expressed in only a subset of the tissues (Figure 4a). No genes were found to be universally expressed *in vivo *but not *in vitro*. As a consequence, the identification of downregulated genes in cell lines depends on the tissue samples present in the comparison. This might explain the lower concordance between different datasets for downregulated genes compared with upregulated genes, as large differences between the types of tissue samples in datasets I and II existed (for example, no tumor samples in dataset II).

The genes downregulated in cell lines and only expressed in subsets of tissues and tumors were likely to represent tissue-specific genes for which the expression was lost in cell lines (Figure 4a). Indeed, examples of tissue-specific genes that were downregulated in cell lines were identified for blood cells (Figure 4, cluster A, for example, PBXIP1, ISGF3 and IkB-alpha), brain tumors (Figure 4, cluster C and sample cluster 6, for example, CCND2 and APPBP2), renal biopsies (Figure 4, cluster E, for example, hMT-If) and brain normal and tumor biopsies (Figure 4, cluster F, for example, Protocadherin 2).

Leukemias (sample clusters 4 and 5 in Figure 4), lymphomas (sample cluster 3 in Figure 4), and germinal center cells (sample cluster 7 in Figure 4) had gene-expression profiles most similar to those of the cell lines. They had downregulated a large portion of the genes similarly downregulated in cell lines (Figure 4, cluster D). They had also upregulation of genes associated with replication (cluster G, for example, TOPII, MCM2, MCM3 and MCM6) and metabolism (cluster H). The information of all genes present in Figure 4 along with its presence in different subclusters can be found in Additional data file 1. A high-resolution image of Figure 4 with all sample names and gene identifiers can be found in Additional data file 3.

### Transcriptional alterations affect multiple biological processes

Because of the considerable and consistent differential expression of genes in cell lines, we used Gene Ontology (GO) to investigate which biological processes were affected by long-term *in vitro *selection and adaptation. Using GoMiner [25] we identified the GO categories over-represented among the differentially expressed genes defined by SAM at a FDR of 1% (735 up- and 1,699 downregulated genes). By this approach, multiple and highly overlapping GO categories showing statistical significance were identified. GoMiner corrects the *p*-values for the multiple comparisons and we set the FDR threshold to 5% for the GO category identification. We found that upregulated genes in cell lines are over-represented for multiple GO categories relating to three main cellular processes: cell cycle; macromolecular biosynthesis, processing, modification and degradation; and energy metabolism (Table 3). Seven genes belonging to the 'histone modification' category were also upregulated. Interestingly, among the downregulated genes we identified many genes involved in 'cell adhesion', 'cell-cell adhesion', 'enzyme linked receptor protein signaling pathway', and 'cell-cell signaling' (Table 4). A similar pattern of downregulated genes involved in cell-cell communication, membrane signaling and second messenger signaling was observed in dataset II (data not shown). We also identified many downregulated genes involved in immune-system functions and antigen presentation. However, these differences were dataset dependent and not observed in dataset II. Therefore these categories were excluded from Table 4 but are given in Additional data file 4.

## Discussion

The use of immortalized cell lines as model systems of normal and pathological tissues is controversial [5,26-28]. There are obvious general differences between the environment of cells growing *in vitro *and that of *in vivo *tissue cells, including oxidative pressure, nutrient accessibility, cell-cell contact and interactions with ECM, as well as in growth rate. These differences influence the gene expression and the phenotype of the cells grown *in vitro*. Many gene-expression studies have analyzed the differences between cell lines derived from a specific tumor tissue to the corresponding tumor tissues and primary cultures [2,10,12,29]. These studies are important to asses how cell-line model systems have maintained the gene expression of their tumor origins, that is, their tissue identities. We have previously developed a method to assess how gene expression in individual cell lines relates to tumors of different tissue origins [30]. It is, however of equal importance to pinpoint the cellular processes affected by long term *in vitro *growth irrespectively of tissue origin. Therefore we have performed a comprehensive analysis of gene-expression profiles of 60 cell lines and 311 samples from multiple tissue origins. The analyses showed that approximately 30% of the genes investigated were differentially expressed in immortalized cell lines.

We used GO to characterize the cellular processes that were transcriptionally altered in cell lines. This analysis identified the common biological processes that were transcriptionally altered in rapidly dividing cells, that is, a molecular portrait of proliferation. In support of previous findings [2,10], these data confirmed an upregulation of genes involved in translation, cell-cycle regulation and DNA replication. In addition, this comparison identified many other cellular processes that were upregulated (Table 2). Genes involved in energy metabolism, nucleotide metabolism, splicing, protein modifications and degradation, and chromatin regulation were enriched among the upregulated genes *in vitro*. As expected, many of the upregulated genes seem to be directly involved in cell divisions. For example, the maintenance methylation enzyme, DNA methyltransferase 1 (DNMT1), was consistently upregulated in the rapidly dividing cell lines. DNMT1 methylates newly synthesized DNA and is directly involved in the DNA replication process. The *de novo *DNA methylation enzymes DNMT3A and DNMT3B were not, however, upregulated in cell lines. Therefore, it is tempting to speculate that the list of upregulated genes is enriched in genes directly involved in the essential cellular processes for rapidly diving cells (for example, DNA replication). The gene list might therefore be used to predict which cellular factors are general and which factors have more specialized regulatory roles. Certain histone-modifying proteins (HDAC1, EZH2, and HP1 beta and gamma subunits) were upregulated in cell lines whereas others were not. Could these factors also be directly involved in DNA replication?

Among the genes downregulated *in vitro *we detected many involved in cell communication, membrane signaling, and adhesion to ECM. A downregulation of genes involved in ECM interactions were previously found in a serial analysis of gene expression (SAGE) study [31]. Our results confirm their observation. We further demonstrate that additional membrane signaling proteins, working downstream of G-protein-coupled receptors, were downregulated *in vitro*. The downregulation of many proteins involved in membrane signaling, cell-cell communication and adhesion to ECM probably reflect the altered environment for cells growing *in vitro *and in defined cell-culture media and in contrast to the organization of cells in tissues [6,26,27]. Indeed, when transplanting tumor cell lines into immunodeficient mice and analyzing the resulting tumors, genes involved in ECM and cell adhesion were again upregulated [32]. The gene-expression comparison presented in this study could also be used for detailed characterization of particular pathways [14] to identify which are up- or downregulated as part of the cell-line adaptation to *in vitro *conditions.

This study compared immortalized cell lines to solid tumors of diverse origins. Tissues are complex, heterogeneous mixtures of cell types, whereas cell lines contain just one more-or-less clonal cell type, selected for its ability to grow under *in vitro *conditions. It is likely that the expression of genes in tumor-derived cell lines is more similar to that in the malignant cells within the tumor tissue. Thus the *in vitro *signature is a combined effect of *in vitro *adaptation and selection for subtypes of cells from the tissue. Although at present it would be methodologically very hard to establish the contribution from either of these two phenomena, some general remarks can be made. Genes more highly expressed in the malignant cell would appear upregulated in cell lines as a result of the enrichment of this cell in culture. Because the tumor samples contained at least 50% malignant cells (usually more, see Materials and methods) this 'enrichment effect' could never result in an artificial fold-change of more than 2. In our data, 344 genes (dataset I) and 1,159 genes (dataset II) were upregulated in cell lines with a fold-change exceeding 2. It is therefore impossible that the enrichment effect explains the major part of the observed upregulation of genes *in vitro*. It could only bias the numbers to a limited extent. On the other hand, the degrees of infiltration of stromal cells vary between different solid tumors [33]. There is a possibility that genes upregulated in stromal cells appear downregulated in cell lines as a result of the lack of these cells in culture. This dilution effect could potentially result in an apparent downregulation in cell lines of genes with a fold-change value exceeding 2. This requires that there is a sixfold change in the expression in the stromal compartment comprising 20% of the cells in the tumor, for a gene to appear downregulated by more than twofold in cell lines. One extreme, but interesting, possibility would be that the cells growing *in vitro *are derived from a putative 'cancer stem cell' [34]. In that case the enrichment effect could be profound, and the observed expression signature would then be a combination of the *in vitro *adaptation and selection for a common cancer stem cell signature. These intriguing issues might be resolved using laser-capture microdissection [35] on specific subpopulations of cells within the tumor for cases where reliable stem-cell markers can be established or applying tissue modeling in *in vitro *three-dimensional culture systems [26,27]. It must be emphasized, however, that the tumor tissue phenotype is very much dictated by the interplay between different cell types, which is decisively interrupted by growth *in vitro *[28,33]. The interplay between malignant cells and stroma can be dissected using xenografts. In a recent study, human cell lines were injected into mice and the effect of stromal components on the gene expression of the malignant cell was specifically investigated [32]. Finally, it is of fundamental importance to pinpoint the common transcriptional differences and similarities of these cell lines to their tissues of origin irrespective of their causes, as in our study. These cell lines are routinely used as model systems of tumors and normal tissues. Therefore the nature and volume of effects related to *in vitro *culture are profoundly relevant.

It would be interesting to investigate the temporal aspects of the establishment of the *in vitro *signature. In a recent study 6- and 24-hour primary cultures of hepatocytes were compared to liver tissues [36]. Not surprisingly, it was found that the gene-expression profiles separated gradually with time. However, the genes reported to be upregulated at 6 and 24 hours are not the same as the ones that were found to be universally upregulated in our tumor-derived cell lines, indicating the need for a longer period of time before the *in vitro *signature gets established. Other studies have identified higher expression of a limited set of proliferation-associated genes in immortalized cancer cell lines when compared with primary cultures [10,29]. Therefore, it is likely that the extensive differential expression observed in this study occur as a result of long-term adaptation due to *in vitro *selection and adaptation.

This study also introduced a fruitful cross-site approach for quantitative comparison of gene-expression data from different laboratories. The growing wealth of gene-expression data available in public databases offers great opportunities for computational experiments. It must, however, be emphasized that a successful comparison of gene-expression data from different laboratories depends on the quality of the data and similarities in the experimental protocols used [37]. Therefore, careful quality controls and validations of gene-expression comparisons must always be performed. If available, raw data files (that is, CEL files) would enable additional quality controls (such as checking the image for hybridization scratches) and the use of different methods to estimate transcript levels [38]. We developed a quality-control procedure by examining the scalar factors, correlation between similar samples, SVD, and an independent validation dataset. This approach was successful in the analysis of gene-expression data from three different laboratories (using the same Affymetrix Hu6800 platform). Thus, quantitative comparisons of gene-expression data from different sites may be feasible.

## Conclusion

This cross-site comparison of gene expression in cell lines, normal, and tumor tissues revealed a distinct *in vitro *gene-expression signature. This signature deserves attention as a biological phenomenon itself, as it can elucidate and teach us about the impressive consequences of *in vitro *selection and adaptation, with implications for tissue organization and future tissue engineering *in vitro*.

## Materials and methods

### Gene-expression data

We compiled gene-expression data on cell lines, normal, and tumor samples from three different studies [15-17] that all used Affymetrix Hu6800 arrays. The National Cancer Institute NCI60 cell-line gene-expression data [15] were downloaded from Cancer Program Data Sets [39]. The tab-delimited text file (NCI60_aug99_resfile.txt) contained scaled expression data together with 'absolute calls' (absent, present and marginal). The 60 cell lines came from the following tissues: lung (*n *= 9), colon (*n *= 7), breast (*n *= 8), ovary (*n *= 6), leukemia (*n *= 6), renal (*n *= 8), melanoma (*n *= 8), prostate (*n *= 2), nervous system (*n *= 6). Gene-expression data for 59 human tissue samples [17] were downloaded from Human Gene Expression Index [40] in an already normalized format and represented the following samples: blood (*n *= 1), brain (*n *= 11), breast (*n *= 2), colon (*n *= 1), cervix (*n *= 1), endometrium (*n *= 2), esophagus (*n *= 1), kidney (*n *= 6), liver (*n *= 6), lung (*n *= 6), muscle (*n *= 6), myometrium (*n *= 2), ovary (*n *= 2), placenta (*n *= 2), prostate (*n *= 4), spleen (*n *= 1), stomach (*n *= 1), testis (*n *= 1), vulva (*n *= 3). Gene-expression profiles of 60 normal and 189 tumor samples from 14 different tissue origins [16] were downloaded as raw (unscaled) gene-expression data (GCM_Total.res) from Cancer Program Data Sets [39]. Tumor tissue origins were: breast, prostate, lung, colon, lymphoma, melanoma, bladder, uterus, leukemia, kidney, ovary, mesothelioma, and central nervous system. Normal samples were from the following tissues: breast, prostate, lung, colon, germinal center, bladder, uterus, peripheral blood, kidney, pancreas, ovary and central nervous system. All tumors were biopsy specimens from primary sites, obtained before any treatment and enriched for at least 50% malignant cells [16]. For further details see [16].

An independent validation dataset (dataset II) that contained both *in vivo *samples (*n *= 70) and cell lines (*n *= 25) hybridized to Affymetrix HGU95A arrays [18] was downloaded from the Gene Expression Atlas [41]. The gene-expression data had previously been scaled using the GeneChip Global Scaling algorithm to a target intensity of 200.

Three datasets were used to assess our ability to classify samples into either cell lines or tissues. Dataset III comprised 10 cell lines and 123 tissue samples [8]. Genes were matched between U133A and HGU95A on the basis of best-match spreadsheets from Affymetrix NetAffx [42]. Dataset IV [24] comprised 15 cell lines and 64 tumors (mostly lymphomas) [24]. Dataset V comprised 10 cell lines and 81 lung tumors and normal biopsies [12] and we used UniGene identifiers to map their genes to our Affymetrix array identifiers. Only a limited number of genes (*n *= 36) of the 576 had a UniGene match. Nevertheless, using only 36 genes most samples were correctly classified as cell lines or tissues. The HUVEC cells of unknown passage from dataset II and FACS-purified cells were excluded from this classification of cell lines and tissues.

### Normalization

To compare the gene-expression data generated in different laboratories we rescaled each sample to equal global chip intensity. The global scaling algorithm was calculated from the positive average difference values excluding the top and bottom 2% average difference values. A reference sample (lung-derived cell line: NSCLC_H460) was chosen on the basis of its average percent present and its average global chip intensity before rescaling. All other samples were rescaled to the equal average chip intensity as the reference sample. We thereafter 'thresholded' the data using a ceiling of 16,000 units and a floor of 20 units.

### Singular value decomposition

Singular value decomposition (SVD) is a standard method in linear algebra and the mathematical details of SVD for gene-expression analysis have been described in detail elsewhere [19-21]. In brief, a gene-expression matrix (with rows of genes and columns of arrays) after SVD is decomposed into three matrices USV^T^. The left singular vectors (hereafter called eigenarrays) are the columns of matrix U, the diagonal in S are the singular values and the rows of V^T ^the right singular vectors. We projected the gene-expression pattern of each sample into a two-dimensional SVD subspace, by measuring the correlation between the gene expression of each sample to the first two eigenarrays. Before SVD calculation we pre-processed the expression data for each gene independently to an average expression level of zero and a standard deviation of one. We used the SVD implementation in Numerical Python (version 23.1) for Python 2.3.3.

### Significance analysis of microarrays

We used the significance analysis of microrrays (SAM) [22] available as an Excel add-in (version 1.21) to identify the number of differentially expressed genes, as a function of the false discovery rate (FDR). We identified statistically significant genes at estimated FDR of zero and 1% (based on 1,000 permutations) and using a fold-change cutoff of 1.5.

### Classification of gene-expression profiles

We used the genes identified as differentially expressed in dataset I and II (*n *= 576) to assess whether we could classify samples in five different datasets into either 'cell lines' or 'tissues'. Dataset I and II correspond to the datasets detailed above (table 1) and were used as initial controls. Before calculation we pre-processed the expression data for each gene independently to an average expression level of zero and a standard deviation of one for each dataset separately. For each dataset, we then calculated the mean gene-expression levels for each gene independently across all cell lines and tissues, respectively. The average cell line expression profile and tissue profile within each dataset were referred to as the 'cell line centroid' and 'tissue centroid'. Then we calculated the Euclidean distance (D_e_) between each sample and the cell line centroid and tissue centroid, respectively. We integrated the two distances into a simple score by calculating the difference between the Euclidean distance to the tissue centroid and cell line centroid. Thus, samples that resemble cell lines more than tissues would have a short Euclidean distance towards the cell line centroid and a longer distance towards the tissue centroid and therefore get a positive score. For all datasets a bimodal distribution of scores was observed (see Additional data file 2 for the distributions of scores for samples in the five datasets). We defined a threshold for each dataset that gave equal amounts of false positives and false negatives. Then all scores above threshold were classified as 'cell line' and all scores below threshold as 'tissue'. The performance of the classification was reported as the accuracy, that is, the sum of the true positives and true negatives divided by the total number of predictions for each dataset.

### GO analysis

We used GoMiner [25] to analyze the lists of up- and downregulated genes for GO categories that were significantly statistically over-represented. We used the second generation GoMiner program that first estimates the *p*-value using Fisher's exact test and then corrects the *p*-values for the multiple comparisons by estimating the FDR. We reported only GO categories that had corrected *p*-values of less than 0.05.

## Additional data files

The following additional data are available with the online version of this paper. Additional data file 1 lists the genes found to be differentially expressed in cell lines versus tissues in both datasets, with corresponding gene names, probe identifiers, SAM *d *scores and fold-change values. The order of the genes in this table is identical to Figure 4. Additional data file 2 contains a figure with a graph of the distribution of scores for all samples in the five different datasets respectively. Additional data file 3 is a high-resolution image of Figure 4 in which all sample names and gene identifiers can be found. Additional data file 4 lists the dataset-specific GO categories downregulated in only cell lines from dataset I. These categories were mainly of immunological processes and are listed with corresponding statistics and GO identifiers. Additional data file 5 describes the calculations used in the discussion to estimate cell composition effects on gene-expression comparisons.

## Supplementary Material

Additional Data File 1 Corresponding gene names, probe identifiers, SAM d scores and fold change values are given. The order of the genes in this table is identical to Figure 4. Click here for file

Additional Data File 2 A figure with a graph of the distribution of scores for all samples in the five different datasets respectively. Click here for file

Additional Data File 3 A high-resolution image of Figure 4 in which all sample names and gene identifiers can be found. Click here for file

Additional Data File 4 These categories were mainly of immunological processes and are listed with corresponding statistics and GO identifiers. Click here for file

Additional Data File 5 A description of the calculations used in the discussion to estimate cell composition effects on gene-expression comparisons. Click here for file

## Figures and Tables

**Table 1:** Sources of gene-expression data

Source	Number of cell lines	Number of normal tissue samples	Number of tumor samples	Dataset	Platform
[15]	60	-	-	I	Hu6800
[17]	-	59	-	I	Hu6800
[16]	-	60	189	I	Hu6800
[18]	25	65	5	II	HGU95A

**Table 2:** Classification of cell lines and tissue samples across five datasets

Dataset reference	Accuracy (%)	Number of cell lines	Number of tissue samples
Dataset I	99*	60	371
Dataset II	100	25	70
Dataset III [8]	100	10	123
Dataset IV [24]	95	15	64
Dataset V [12]	96	10	81

*One cell line (breast cell line HS578T) was misclassified as a tissue sample.

**Table 3:** Biological process upregulated *in vitro*

GO category	Total number of genes	Genes changed	Log_10 _(*p*-value)	FDR	GO ID
**Translation**					
Translation	76	36	-7.95	0.0000	GO:0043037
Ribosome biogenesis and assembly	42	19	-4.10	0.0037	GO:0042254
Ribosome biogenesis	41	19	-4.27	0.0000	GO:0007046
Regulation of translation	33	14	-2.81	0.0077	GO:0006445
Translational initiation	23	13	-4.20	0.0042	GO:0006413
tRNA metabolism	27	12	-2.69	0.0070	GO:0006399
tRNA modification	23	11	-2.82	0.0078	GO:0006400
tRNA aminoacylation for protein translation	21	10	-2.59	0.0125	GO:0006418
tRNA aminoacylation	21	10	-2.59	0.0125	GO:0043039
rRNA processing	17	10	-3.53	0.0056	GO:0006364
rRNA metabolism	17	10	-3.53	0.0056	GO:0016072
Regulation of translational initiation	14	8	-2.79	0.0075	GO:0006446
Translational elongation	14	7	-2.07	0.0400	GO:0006414
Transcription from Pol I promoter	7	5	-2.44	0.0141	GO:0006360
					
**Splicing**					
RNA processing	123	52	-9.02	0.0000	GO:0006396
RNA metabolism	130	52	-8.00	0.0000	GO:0016070
mRNA metabolism	64	21	-2.27	0.0217	GO:0016071
mRNA processing	57	20	-2.56	0.0123	GO:0006397
RNA splicing	41	18	-3.70	0.0030	GO:0008380
RNA splicing, via transesterification reactions with bulged adenosine as nucleophile	33	15	-3.37	0.0050	GO:0000377
RNA splicing, via transesterification reactions	33	15	-3.37	0.0050	GO:0000375
Nuclear mRNA splicing, via spliceosome	33	15	-3.37	0.0050	GO:0000398
RNA modification	25	11	-2.46	0.0143	GO:0009451
					
**Nucleotide metabolism**					
Nucleobase, nucleoside, nucleotide and nucleic acid metabolism	806	192	-4.43	0.0000	GO:0006139
Nucleotide metabolism	61	20	-2.18	0.0304	GO:0009117
Nucleotide biosynthesis	45	16	-2.21	0.0303	GO:0009165
Ribonucleotide metabolism	28	13	-3.09	0.0047	GO:0009259
Ribonucleotide biosynthesis	27	13	-3.28	0.0048	GO:0009260
Purine nucleotide metabolism	29	12	-2.37	0.0164	GO:0006163
Purine nucleotide biosynthesis	26	12	-2.86	0.0080	GO:0006164
Purine ribonucleotide metabolism	25	11	-2.46	0.0143	GO:0009150
Purine ribonucleotide biosynthesis	24	11	-2.63	0.0115	GO:0009152
Nucleoside triphosphate metabolism	23	10	-2.23	0.0299	GO:0009141
Ribonucleoside triphosphate metabolism	20	9	-2.17	0.0295	GO:0009199
Ribonucleoside triphosphate biosynthesis	19	9	-2.35	0.0167	GO:0009201
Nucleoside triphosphate biosynthesis	20	9	-2.17	0.0295	GO:0009142
Purine ribonucleoside triphosphate metabolism	20	9	-2.17	0.0295	GO:0009205
Purine ribonucleoside triphosphate biosynthesis	19	9	-2.35	0.0167	GO:0009206
Purine nucleoside triphosphate metabolism	21	9	-2.00	0.0413	GO:0009144
Purine nucleoside triphosphate biosynthesis	19	9	-2.35	0.0167	GO:0009145
Nucleoside metabolism	14	7	-2.07	0.0400	GO:0009116
					
**Protein modifiication and degradation**					
Protein metabolism	836	210	-6.86	0.0000	GO:0019538
Protein biosynthesis	207	72	-7.76	0.0000	GO:0006412
Intracellular transport	176	63	-7.36	0.0000	GO:0046907
Protein transport	149	52	-5.75	0.0000	GO:0015031
Intracellular protein transport	138	50	-6.11	0.0000	GO:0006886
Amino acid and derivative metabolism	126	36	-2.31	0.0198	GO:0006519
Amino acid metabolism	98	29	-2.19	0.0297	GO:0006520
Ubiquitin-dependent protein catabolism	48	26	-7.39	0.0000	GO:0006511
Modification-dependent protein catabolism	48	26	-7.39	0.0000	GO:0019941
Protein targeting	70	23	-2.44	0.0139	GO:0006605
Protein folding	46	22	-5.14	0.0000	GO:0006457
Ubiquitin cycle	31	12	-2.10	0.0351	GO:0006512
Amino acid activation	21	10	-2.59	0.0125	GO:0043038
Polyamine metabolism	5	4	-2.27	0.0271	GO:0006595
					
**Metabolism**					
Metabolism	2008	457	-12.88	0.0000	GO:0008152
Biosynthesis	423	119	-6.33	0.0000	GO:0009058
Energy pathways	128	38	-2.74	0.0074	GO:0006091
Energy derivation by oxidation of organic compounds	89	32	-4.02	0.0036	GO:0015980
Main pathways of carbohydrate metabolism	56	20	-2.67	0.0069	GO:0006092
Coenzyme and prosthetic group metabolism	55	18	-2.00	0.0419	GO:0006731
Coenzyme metabolism	44	16	-2.32	0.0200	GO:0006732
Glucose catabolism	30	12	-2.23	0.0307	GO:0006007
Coenzyme and prosthetic group biosynthesis	31	12	-2.10	0.0351	GO:0046138
Oxidative phosphorylation	13	11	-6.25	0.0000	GO:0006119
Coenzyme biosynthesis	23	10	-2.23	0.0299	GO:0009108
Cellular respiration	11	9	-4.94	0.0000	GO:0045333
Aerobic respiration	9	8	-4.92	0.0000	GO:0009060
Tricarboxylic acid cycle	18	8	-1.94	0.0462	GO:0006099
ATP synthesis coupled electron transport (*sensu *Eukarya)	6	5	-2.91	0.0061	GO:0042775
ATP synthesis coupled electron transport	6	5	-2.91	0.0061	GO:0042773
					
**Cell-cycle progression**					
Cell cycle	324	89	-4.32	0.0000	GO:0007049
Cell organization and biogenesis	315	83	-3.38	0.0054	GO:0016043
DNA metabolism	188	64	-6.53	0.0000	GO:0006259
Mitotic cell cycle	153	58	-7.84	0.0000	GO:0000278
Cytoplasm organization and biogenesis	202	55	-2.73	0.0073	GO:0007028
DNA replication and chromosome cycle	83	30	-3.85	0.0033	GO:0000067
M phase	62	26	-4.68	0.0000	GO:0000279
Nuclear organization and biogenesis	79	25	-2.36	0.0176	GO:0006997
DNA packaging	69	25	-3.31	0.0049	GO:0006323
S phase of mitotic cell cycle	72	25	-3.00	0.0043	GO:0000084
Chromosome organization and biogenesis (*sensu *Eukarya)	77	24	-2.20	0.0300	GO:0007001
DNA replication	67	23	-2.72	0.0071	GO:0006260
Nuclear division	54	22	-3.82	0.0031	GO:0000280
Establishment and/or maintenance of chromatin architecture	64	21	-2.27	0.0217	GO:0006325
M phase of mitotic cell cycle	45	20	-4.15	0.0040	GO:0000087
DNA repair	59	20	-2.36	0.0173	GO:0006281
Mitosis	42	19	-4.10	0.0037	GO:0007067
Microtubule-based process	45	19	-3.61	0.0059	GO:0007017
DNA-dependent DNA replication	35	15	-3.04	0.0044	GO:0006261
Microtubule cytoskeleton organization and biogenesis	27	14	-3.94	0.0034	GO:0000226
G1/S transition of mitotic cell cycle	35	13	-2.06	0.0396	GO:0000082
G2/M transition of mitotic cell cycle	21	9	-2.00	0.0413	GO:0000086
M-phase specific microtubule process	12	7	-2.56	0.0132	GO:0000072
Chromosome segregation	14	7	-2.07	0.0400	GO:0007059
Microtubule nucleation	9	6	-2.65	0.0117	GO:0007020
DNA replication initiation	10	6	-2.32	0.0190	GO:0006270
Spindle assembly	8	6	-3.05	0.0045	GO:0007051
Tubulin folding	9	6	-2.65	0.0117	GO:0007021
Mitotic spindle assembly	6	5	-2.91	0.0061	GO:0007052
Pre-replicative complex formation and maintenance	5	4	-2.27	0.0271	GO:0006267
					
**Chromatin modifications**					
Histone modification	12	7	-2.56	0.0132	GO:0016570
Covalent chromatin modification	12	7	-2.56	0.0132	GO:0016569
					
**Others**					
Physiological process	2917	574	-3.84	0.0032	GO:0007582
Macromolecule biosynthesis	345	100	-5.98	0.0000	GO:0009059
Response to endogenous stimulus	77	23	-1.89	0.0486	GO:0009719
Response to DNA damage stimulus	71	22	-2.02	0.0412	GO:0006974

**Table 4:** Biological process downregulated *in vitro*

GO category	Total number of genes	Genes changed	Log_10 _(*p*-value)	FDR	ID
Membrane signaling and cell adhesion					
Cell communication	1088	565	-13.76	0.0000	GO:0007154
Signal transduction	831	428	-8.86	0.0000	GO:0007165
Cell surface receptor linked signal transduction	413	232	-8.66	0.0000	GO:0007166
Cell adhesion	257	139	-4.10	0.0000	GO:0007155
Cell-cell signaling	240	132	-4.38	0.0000	GO:0007267
Cell motility	197	105	-2.91	0.0171	GO:0006928
G-protein coupled receptor protein signaling pathway	175	102	-4.87	0.0000	GO:0007186
Enzyme linked receptor protein signaling pathway	107	61	-2.78	0.0231	GO:0007167
Cell-cell adhesion	87	53	-3.41	0.0000	GO:0016337
G-protein signaling, coupled to IP3 second messenger (phospholipase C activating)	35	23	-2.32	0.0490	GO:0007200
Extracellular structure organization and biogenesis	17	14	-3.02	0.0029	GO:0043062
Extracellular matrix organization and biogenesis	16	13	-2.73	0.0225	GO:0030198
