# Supplementary material for: The molecular portrait of in vitro growth by meta-analysis of gene-expression profiles
Source: Genome Biol. 2005 Jul 27;6(8):R65. doi: 10.1186/gb-2005-6-8-r65 (PMC1273632; doi:10.1186/gb-2005-6-8-r65)
Supplement: Additional Data File 2 — A figure with a graph of the distribution of scores for all samples in the five different datasets respectively. [file gb-2005-6-8-r65-S2.pdf]

Additional data file 2

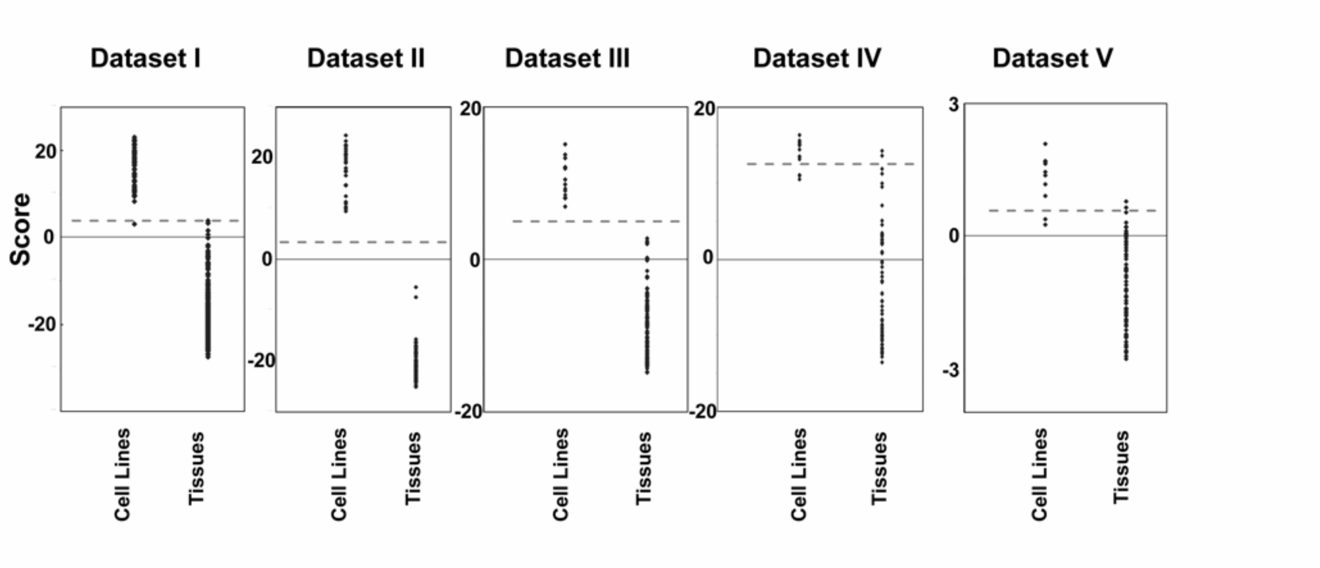

Classification of cell lines and tissue samples across 5 datasets based upon their expression of the differentially expressed genes identified in dataset I and II. The difference in Euclidean distance (score) for each sample dataset respectively is presented and the samples within each dataset was stratified for the true cell lines and tissue samples (x-axis categories). The dashed line represent the objective classification thresholds used that gives equal number of false positives and false negatives. For details upon classification see materials and methods.
