# Supplementary figures and images for: The molecular portrait of in vitro growth by meta-analysis of gene-expression profiles
Source: Genome Biol. 2005 Jul 27;6(8):R65. doi: 10.1186/gb-2005-6-8-r65 (PMC1273632; doi:10.1186/gb-2005-6-8-r65)

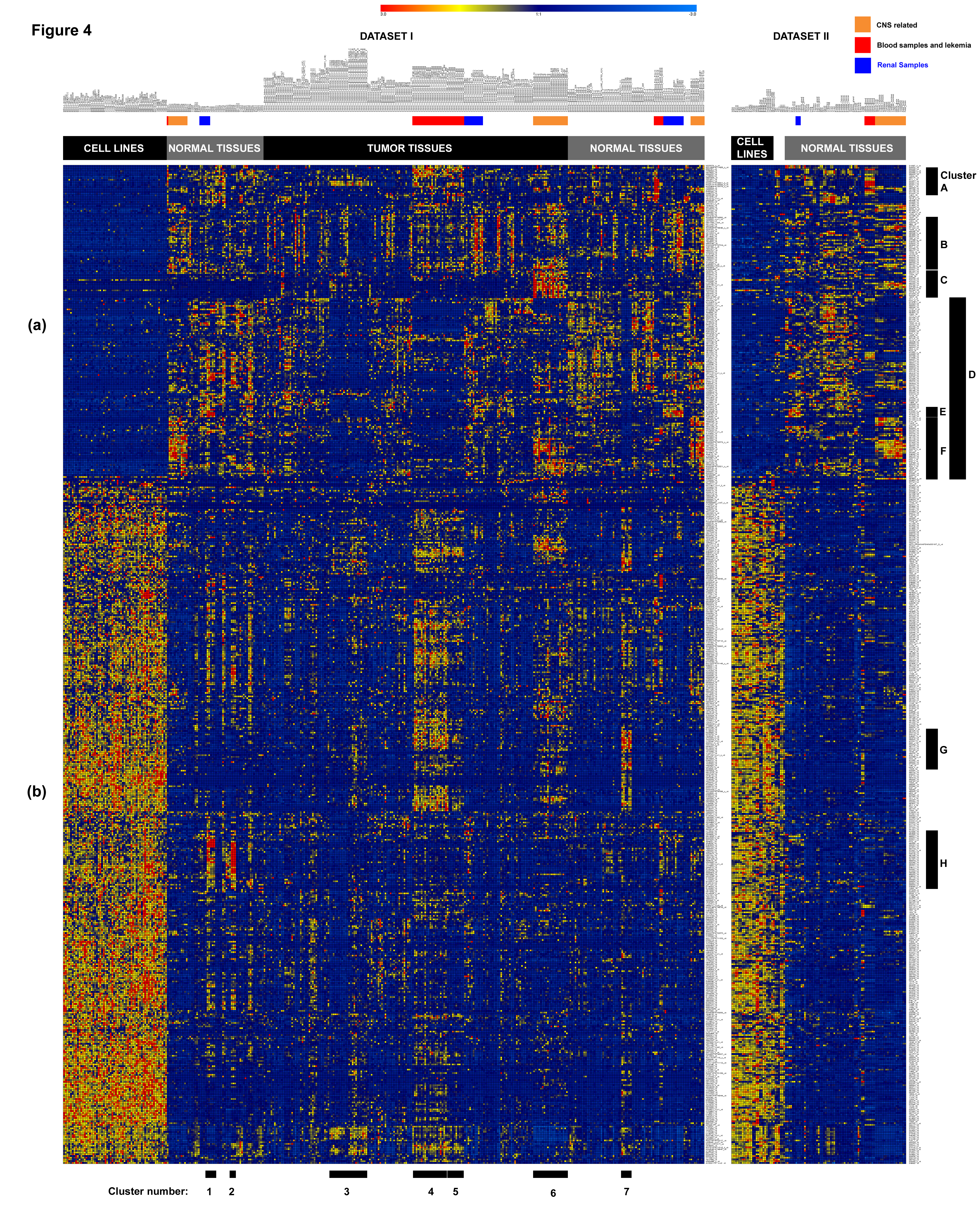

Supplement: Additional Data File 3 — A high-resolution image of Figure 4 in which all sample names and gene identifiers can be found. [file gb-2005-6-8-r65-S3.png]
