# Supplementary material for: The molecular portrait of in vitro growth by meta-analysis of gene-expression profiles
Source: Genome Biol. 2005 Jul 27;6(8):R65. doi: 10.1186/gb-2005-6-8-r65 (PMC1273632; doi:10.1186/gb-2005-6-8-r65)
Supplement: Additional Data File 5 — A description of the calculations used in the discussion to estimate cell composition effects on gene-expression comparisons. [file gb-2005-6-8-r65-S5.doc]

**Additional Data File 5:** A simplified model of the effects of cell-type composition on comparisons between cell lines and tumor tissues.

A cell lines is assumed to be consisting of only the malignant cell type, here called A-cells.

A tumor tissue is assumed to be consisting of malignant cells, A-cells, and additional stroma and bystander cells, B-cells. The percentages of A-cells and B-cells within a tumor tissue are Y% A-cells and consequently 100-Y% B-cells.

The gene expression level of a gene in the respective cell types is denoted Xa and Xb. Then the average expression in cell lines, which is composed of 100% A-cells, is 100 * Xa.

In a tumor tissue, the average gene expression is, Y * Xa + (100-Y) * Xb.

*Enrichment effect:*

The ‘enrichment effect’ concerns the enrichment of malignant cells in immortalized cell lines. A gene that is more highly (or only) expressed in the malignant cells could appear to have a higher average expression level in cell lines only due to the enrichment of these cells in cell cultures.

Since, all tumor tissues used in the study consisted of at least 50% malignant cells [16], Y is at least 50%. When comparing cell lines to tumor tissues, the fold change in expression level of a gene can be formalized as:

Fold Change = 100*Xa / [ Y*Xa + (100-Y)*Xb ], with the above constraint that Y > 50.

From the equation it is evident that no matter how much a gene would be up-regulated in the malignant cell, the fold change can never exceed two, since at least 50% of the cells within the tumor tissue will express the same amount of the gene. If a gene is exclusively expressed in malignant (A-cells) and the tumor is only consisting of 50% malignant cells, then and only then, would a fold change of two appear as an result of the ‘enrichment effect’.

*Dilution effect*

There is a possibility that genes up-regulated in stromal cells appear down-regulated in cell lines due to the lack of these cells in culture. This ‘dilution effect’ could potentially result in an apparent down-regulation in cell lines. Again, using a simplified model we can get some rough estimates on how an up-regulation in a specific stroma cell would affect cell line to tissue comparisons.

For example, for a gene with expression levels Xa in malignant cells. If the gene is up-regulated 6-times (6*Xa) in a particular cell comprising 20% of the total tumor, then the total expression in the tumor would be 20*6*Xa + 80*Xa. In the cell lines the expression would simply be 100*Xa and the fold change: 100*Xa / [20*6*Xa + 80*Xa] = 100*Xa / 200*Xa, i.e. 2.

For a gene to appear down-regulated in cell lines with a fold change exceeding 2, it requires that there is a 6-fold expression up-regulation in the stromal compartment comprising 20 % of the cells in the tumor, for a gene to appear down-regulated by more than two fold in cell lines.
